# Sailing between Scylla and Charybdis: invited response to ‘Blame or discovery?’

**DOI:** 10.1192/bjb.2023.40

**Published:** 2024-04

**Authors:** Claire Hilton

**Keywords:** History of psychiatry, historical inquiry, hagiographic representations, obituaries, psychiatrists

## Abstract

This brief commentary reflects on navigating two dangers of historical research into psychiatry: hagiographic representations of psychiatrists; and accusations of their self-interest and oppression of vulnerable people.

I would like to focus on two of George Ikkos's comments:^1^ ‘Although fairness is undoubtedly a foundational value in both history and psychiatry, arguably the key driver in historical enquiry should be discovery rather than praise or blame’ and ‘Consistent with her institutional position, Hilton presses repeatedly her legitimate anxiety lest psychiatrists be unfairly criticised or blamed’.

As a historian, I aim to understand and explain the past as impartially and objectively as possible. Historical research is a voyage of discovery, but when seeking out psychiatry and psychiatrists, one needs to navigate cautiously to avoid the dangers of Scylla and Charybdis.

On one side are hagiographic representations: *Festschriften*; biographies; eponymous donations, bequests, lectureships or terminology; obituaries which follow the aphorism *De mortuis nil nisi bonum* (‘Of the dead, nothing but good’). Their messages often originate from psychiatric colleagues and institutions. On the other side, widely quoted historical analyses by Michel Foucault,^2^ Andrew Scull,^3^ Elaine Showalter,^4^ Akinobu Takabayashi^5^ and others have dominated the landscape, often featuring psychiatrists as seeking to benefit themselves while oppressing vulnerable people whom they were meant to be helping.

The polarities of these Scylla and Charybdis sources are hard to reconcile, and we may become enmeshed in either at our peril. Incredulity at what others had written about psychiatry and psychiatrists of the past drew me into researching the subject, well before taking up my ‘institutional position’.

Steering cautiously and critically with an open mind as to what one might find are prerequisites for a successful voyage of historical exploration. ‘Praise and blame’ about past generations are by-products of historical research, not motivations or goals for it. Understanding what happened in the past, however, may contribute to avoiding psychiatry and psychiatrists being ‘unfairly criticised or blamed’ in the future.

## Data Availability

Data availability is not applicable to this article as no new data were created or analysed in this study.

## About the author

**Claire Hilton**, FRCPsych, FRHistS, is historian in residence at the Royal College of Psychiatrists and an Honorary Research Fellow at Birkbeck University of London, London, UK.
